# Small Tails Tell Tall Tales – Intra-Individual Variation in the Stable Isotope Values of Fish Fin

**DOI:** 10.1371/journal.pone.0145154

**Published:** 2015-12-15

**Authors:** Brian Hayden, David X. Soto, Tim D. Jardine, Brittany S. Graham, Richard A. Cunjak, Atso Romakkaniemi, Tommi Linnansaari

**Affiliations:** 1 Biology Department, University of New Brunswick, New Brunswick, Canada; 2 Environment Canada, Saskatoon, Saskatchewan, Canada; 3 School of Environmental and Sustainability, Toxicology Centre, University of Saskatchewan, Saskatoon, Saskatchewan, Canada; 4 National Institute of Water and Atmospheric Research (NIWA), Greta Point, Wellington, New Zealand; 5 Natural Resources Institute Finland, Oulu, Finland; Bournemouth University, UNITED KINGDOM

## Abstract

**Background:**

Fish fin is a widely used, non-lethal sample material in studies using stable isotopes to assess the ecology of fishes. However, fish fin is composed of two distinct tissues (ray and membrane) which may have different stable isotope values and are not homogeneously distributed within a fin. As such, estimates of the stable isotope values of a fish may vary according to the section of fin sampled.

**Methods:**

To assess the magnitude of this variation, we analysed carbon (*δ* ^13^C), nitrogen (*δ* ^15^N), hydrogen (*δ* ^2^H) and oxygen (*δ* ^18^O) stable isotopes of caudal fin from juvenile, riverine stages of Atlantic salmon (*Salmo salar*) and brown trout (*Salmo trutta*). Individual fins were sub-sectioned into tip, mid and base, of which a further subset were divided into ray and membrane.

**Findings:**

Isotope variation between fin sections, evident in all four elements, was primarily related to differences between ray and membrane. Base sections were^13^C depleted relative to tip (~ 1 ‰) with equivalent variation evident between ray and membrane. A similar trend was evident in *δ* ^2^H, though the degree of variation was far greater (~ 10 ‰). Base and ray sections were ^18^O enriched (~ 2 ‰) relative to tip and membrane, respectively. Ray and membrane sections displayed longitudinal variation in ^15^N mirroring that of composite fin (~ 1 ‰), indicating that variation in^15^N values was likely related to ontogenetic variation.

**Conclusions:**

To account for the effects of intra-fin variability in stable isotope analyses we suggest that researchers sampling fish fin, in increasing priority, 1) also analyse muscle (or liver) tissue from a subsample of fish to calibrate their data, or 2) standardize sampling by selecting tissue only from the extreme tip of a fin, or 3) homogenize fins prior to analysis.

## Introduction

The application of stable isotope-based approaches to ecological research is growing at a near exponential rate [1]. Stable isotopes are used to examine trophic relationships, physiology and migration history across all levels of biological organisation [2–4]. The stable isotopes of carbon (^13^C) and nitrogen (^15^N) are the most commonly applied in ecological research [1]. As the *δ* ^13^C and *δ* ^15^N values of a consumer are related to the same ratios in their prey, researchers can use these markers to reconstruct the resource use of an organism, predator–prey interactions or even the entire food-web structure [5,6]. In contrast, stable isotopes of hydrogen (deuterium, hereafter ^2^H) can differentiate between allochthonous and autochthonous resource use in freshwater consumers [7,8], and are particularly useful to re-create migration history or to assign point of origin to material, for which they can be used in conjunction with stable isotopes of oxygen (^18^O) [9]. Furthermore as different body tissues turnover at different rates, analysis of the isotope ratios of different tissues can provide an outline of temporal variability in the ecology of an organism [10,11].

One of the principal disadvantages of stable isotope based ecological research is that sampling is often destructive. Muscle is the most commonly analysed animal tissue in these studies and in the majority of cases obtaining a sufficient sample requires the animal to be sacrificed [1,10]. This has obvious ethical implications for the field especially in relation to studies involving rare or threatened species [12,13]. In recent years, the number of studies employing non-lethal sampling has increased. This is most evident in ornithological studies where destructive samples have been replaced with non-lethal sampling of blood or non-invasive collection of feathers. In studies examining fur bearing mammals, hair and vibrissae samples have begun to replace muscle [14–16]. In fish biology, the focus of the current study, scales [17] and fin clips [18,19] represent non-lethal alternatives to commonly sampled muscle and liver[20].

Fish scales are somewhat troublesome, due their heterogeneous structure which characterises the entire lifetime of the fish [21,22]. Fin clips, however, are routinely taken during fish population surveys and their isotopic turnover rate is similar to that of muscle [11,23]. This has led to fin clips being proposed as an alternative to muscle for stable isotope studies of fish [24–27]. However, fish fin is not a homogeneous structure. Fins are composed of bone and cartilage rays interspersed between a thin epithelial skin membrane [28]. These tissues will likely have a different isotopic turnover time and may have different isotope ratios [11]. Furthermore, the relative proportion of ray and membrane components of a fishes’ fin varies from predominantly ray at the base to predominantly membrane at the tip [29]. If such variation exists within fins, it may be sufficient to confound estimates of resource use or movements based on stable isotope analysis of fish fin. Due to the ethical benefits of using fish fin rather than muscle, an understanding of such confounding effects would be of great utility to the field.

To address these issues we assessed the levels of intra-fin variability in four commonly used stable isotopes (*δ* ^13^C, *δ* ^15^N, *δ* ^2^H and *δ* ^18^O) in wild-caught, juvenile anadromous Atlantic salmon (*Salmo salar;* hereafter salmon) and brown trout (*Salmo trutta*, hereafter trout) sampled in the Tornionjoki river system in northern Finland. We formulated the study around three principal hypotheses, which tested the sources of variation within fins: Firstly, as fish fin is not homogenous in structure [29], we predicted (hypothesis 1; H_1_) that the isotopic composition within an individual fin would not be homogenous. The remaining two hypotheses (H_2_ and H_3_) explored the source of variation observed within fins. We predicted (H_2_) that variation within fins was a reflection of the relative abundance of isotopically distinct ray and membrane sections, and that longitudinal isotope variation from the tip to the base sections of the fin would mirror the variation between ray and membrane. An alternate hypothesis (H_3_) related to fin regeneration. Fish, particularly juvenile salmon and trout, reside in turbulent environments. As a consequence their fins are constantly regenerating due to damage and abrasion with the substrate [30]. If a fish changes its diet or location while fins are regenerating this may be reflected as variation in the isotope ratio between base and regenerated tip sections. If this regeneration hypothesis (H_3_) explained the heterogeneous patterns in fish fin we expected that longitudinal isotope variation from tip to base of ray and membrane analysed in isolation would be equivalent to that observed from tip to base sections of composite (ray + membrane) fin.

## Method

### Field sampling

**Fig 1:**
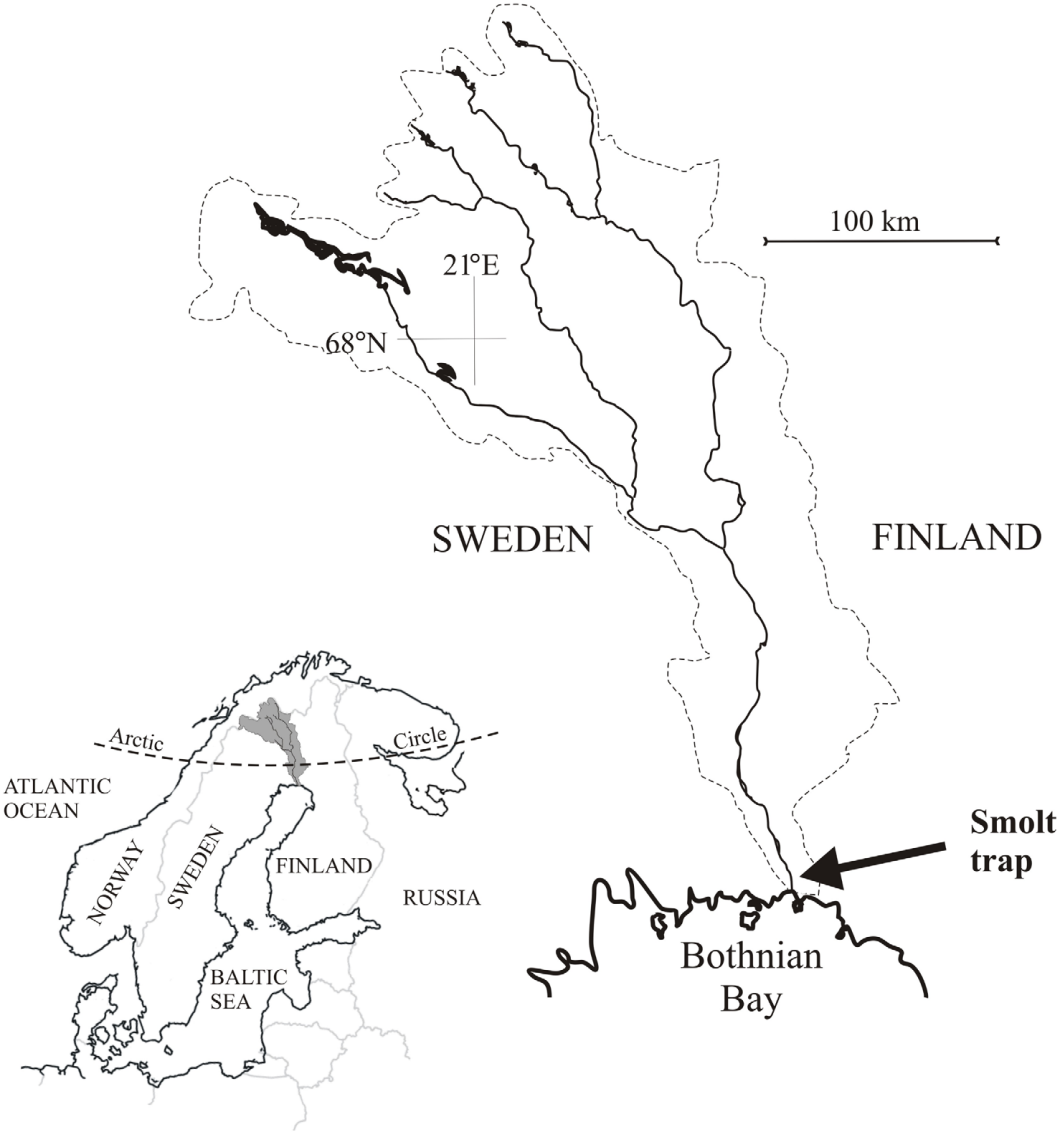
Map of Study Region. The River Tornionjoki drainage forms the northern boundary between Finland and Sweden. The main rivers and the smolt sampling location near the river mouth are highlighted.

The fin samples used in this study were collected as a part of a salmon and trout smolt monitoring program conducted in the River Tornionjoki by the Natural Resources Institute of Finland. The Tornionjoki River system (drainage area 40,000 km^2^) forms a 500-km long border between Finland and Sweden and flows into the Baltic Sea with an annual mean discharge of approximately 380 m^3^ s^-1^ (Fig 1). In the last two decades, the river has had a dramatic increase in returns of adult salmon and is currently one of the most productive salmon rivers in the world, with annual returns exceeding 100,000 adult salmon [31].

Juvenile salmon and trout were sub-sampled in 2006 from the smolt catch of a large fyke-net (100m leader width, set at 2—5m depth in center of 800m wide channel) used when estimating the total smolt run of the Tornionjoki River. The sampling location was approximately 5 km upstream from the river mouth (65°52'23" N; 24° 8'22" E; Fig 1). The total smolt run in 2006 was estimated at approximately 830,000 salmon and 13,000 trout [31]. Salmon (sampling dates from 17 May to 3 July) and trout (sampling dates from 18 May to 13 June) were randomly selected from the smolt catch. Fish were removed from the nets several times per day during the peak smolt run and daily at all other times. Each fish was measured (mm), weighed (0.1g) and a section of caudal fin was collected. Fin samples were frozen (-20°C) within 12 hours of sampling and later oven-dried (60°C; 24-h). Samples were shipped to the Stable Isotopes in Nature Laboratory (New Brunswick, Canada) for further preparation and analysis.

For stable isotope analysis, 108 salmon and 19 trout were selected from this sample. The total length and weight of the analysed salmon ranged from 117 mm to 188 mm (avg. 142 ± 13 mm SD) and from 10.2 g to 48 g (avg. 20.3 ± 6.6 g SD), respectively, with a median age of 3 years. The total length and weight of the analysed trout ranged from 134 mm to 214 mm (avg. 182 ± 19 mm SD) and from 17.6 g to 73.0 g (avg. 48.4 ± 14.5 g SD), respectively, with a median age of 3 years.

### Ethics statement

Permission for sampling was sought and granted under licence from the Lapland County Administrative of the Ministry of Forestry and Agriculture as well as the Finnish-Swedish Transboundary River Commission and informally from private water owners at the sampling site. Salmon and trout are classified as protected fishes in Finland and are subject to a national monitoring program. A portion of this monitoring program consists of lethal sampling of smolts throughout the annual migration period to monitor the sex ratio of the migrating fish. The fin samples for this study required an atypically large section of caudal fin to ensure each sample included base, mid- and tip sections of the caudal fin. All the samples for this study were opportunistically collected from fish that were sacrificed for the purposes of the sex determination as part of a regular national monitoring program and no fish were sacrificed solely for the purposes of this study. The sampling procedure was approved by the Finnish Animal Ethics Committee. Licences and documentation, in Finnish, are available from the authors on request.

### Laboratory analysis

**Fig 2:**
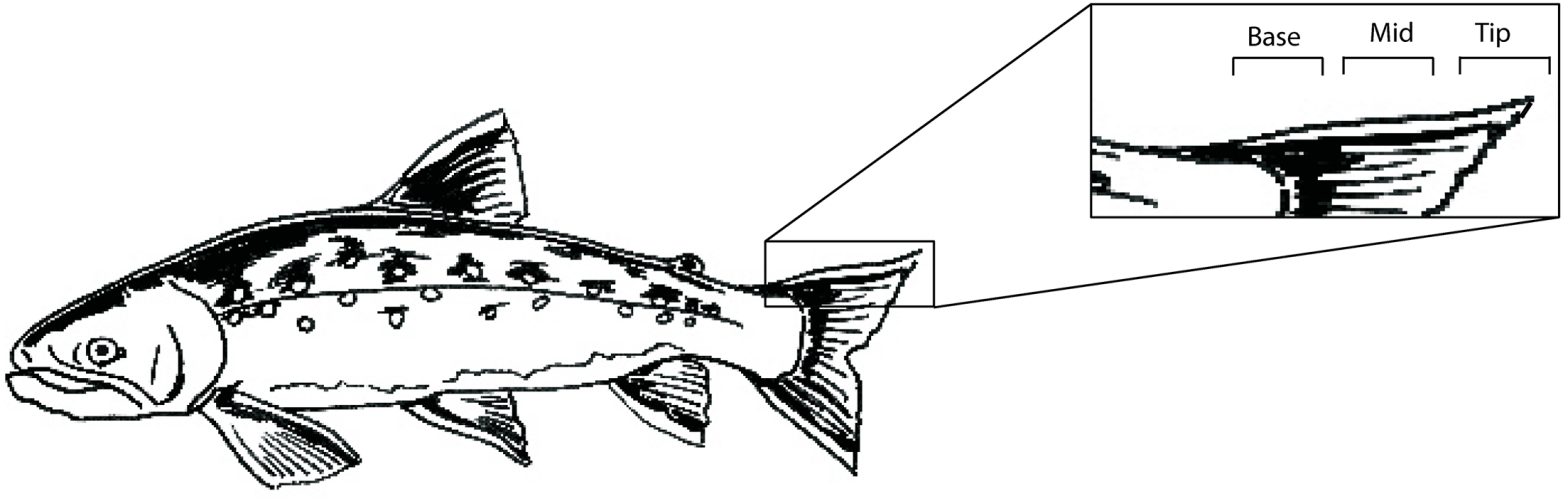
Subsections of fin analysed. Diagram of brown trout highlighting the relative locations of tip, mid and base sections sampled on each fin. Image provided courtesy of P. Antti-Poika.

The fins were sub-sectioned into three groups (Fig 2). The first group (n = 60; Tip-Mid-Base; hereafter TMB) were cut into three equidistant sections representing the distal tip, mid and base section of each fin. To account for the potential biasing effects of lipids within the fins [32,33] we also analysed fins from which lipids had been chemically removed. We assumed that if variation between fin sections was similar in lipid extracted and non-extracted samples then this variation was related to differences in the isotopic composition of the fin rather than a variation in the proportion of lipids within the fin. The lipid extracted group (TMB.LE) were treated with repeated (n = 3) immersion (30 minutes) in a chloroform: methanol (2: 1) solution to remove lipids. Following the final immersion, each fin was dried at 60°C for 24 hours. These lipid-extracted fins (n = 60) were subsequently cut into three equidistant sections as outlined above. The final group (hereafter TMB.RM) were cut into three equidistant sections and each section was further dissected into ray and membrane components. The sample size of trout (n = 19) was too small to facilitate analysis of TMB.LE or TMB.RM groupings for this species. Hence, trout fins were analysed solely as TMB. Sufficient tissue to facilitate all isotope analyses was collected from a few individuals, but generally samples for different experimental groups were obtained from separate fish.

For carbon and nitrogen stable isotope analysis, 1.0 ± 0.1mg subsamples of each fin section were placed in tin-foil cups. Samples were combusted and analyzed in a Delta Plus continuous-flow, isotope-ratio mass spectrometer (Thermo Finnegan GmbH, Bremen, Germany) connected to a Carlo Erba NC2500 elemental analyzer (ThermQuest S.p.A., Milan, Italy). Carbon and nitrogen isotope ratios are reported relative to the international standards Vienna PeeDee Belemnite carbonate (V-PDB) and atmospheric nitrogen (AIR), respectively. Analytical error was calculated as 0.1 ‰ for both *δ* ^13^C and *δ* ^15^N based on repeat analyses of an in-house standard.

For hydrogen and oxygen isotope analysis, 0.2 ± 0.05 mg subsamples of each fin section were placed in silver foil cups. Stable-hydrogen and oxygen isotope measurements for animal tissues were normalized to the international standard VSMOW (Vienna Standard Mean Ocean Water) using standards EC1 and EC2 (Environment Canada, Saskatoon, Canada). We determined the non-exchangeable *δ* ^2^H of samples using the comparative equilibration approach [34] with two secondary standards (EC1 and EC2). These standards were previously calibrated to account for the hydrogen exchangeability between the hydrogen atoms of ambient water vapor and tissues [34,35]. Samples and standards were allowed to exchange with local atmospheric hydrogen for a minimum of 72 hours prior to analysis. Samples were combusted and analysed using a High Temperature Conversion Elemental Analyser (Thermo Scientific GmbH, Bremen, Germany) connected to a Delta *Plus* XP continuous flow isotope-ratio mass spectrometer (Thermo Scientific GmbH, Bremen, Germany). Repeat analysis of an in house standard, keratin (Spectrum-Porcine #SJ1400), indicated that analytical precision was better than 2 ‰ and 0.5 ‰ for *δ* ^2^H and *δ* ^18^O, respectively.

### Data analysis

The stable isotope data associated with this work are provided in the Supporting Information file with this paper (S1 File). The natal and home range of each fish sampled was not known, and as the river basin covers > 40, 000 km^2^ along > 500 km North-South gradient, they likely forage in numerous habitats that encompassed different isotopic baselines. As such, a direct comparison between fish would likely be affected by variation associated with the isotopic baseline of each individual’s home range. To avoid this, we used paired analyses to assess the differences within individuals.

Paired Welch t-tests (a non-parametric equivalent of the paired Student’s t-test) were used to examine the variation in *δ* ^13^C, *δ* ^15^N, *δ* ^18^O, *δ* ^2^H values between sections of each fin. Pairwise comparisons were made between the tip, mid and base sections of each fin to test for heterogeneity in the isotope values within fins from both non-treated (TMB) and lipid-extracted (TMB.LE) salmon and all trout (H_1_). To determine the effect of lipid extraction on intra-fin variability, a Welch t-test was used to compare the difference between tip and base sections of non-treated and lipid-extracted salmon (H_1_). To test H_2_, that the isotope ratios of ray and membrane would differ, pairwise comparisons of the isotope ratios of ray and membrane were conducted across tip, mid and base sections of all TMB.RM fins. Finally, to test H_3_ and determine whether isotopic variation within fins was due to isotopic shifts associated with fin regeneration we used paired Welch t-tests to examine the variation between tip, mid and base sections separately for ray and membrane tissues in TMB.RM group fish.

If the heterogeneity within fins was related to variation between the isotope ratios of ray and membrane we expected that variation observed between ray and membrane would reflect that observed between base and tip sections of composite (ray + membrane) fins. In contrast, if the variation related to fin regeneration then we expected that variation between tip, mid and base sections of ray and membrane independently would mirror that observed in composite fins. A Chi-square test was used to test whether the variation between the isotope ratios of tip and base sections was due to variation between membrane and ray or longitudinal variation within each tissue. Variation between tip and base was classed as tip enriched whereby tip sections were enriched in a specific isotope relative to base sections (option 1), or base enriched, whereby base sections were enriched relative to tip (option 2). The count of options 1 and 2 was then compared with variation between membrane and ray (option 1: membrane enriched, whereby membrane was enriched relative to ray; option 2: ray enriched, whereby ray was enriched relative to membrane), tip and base of fin membrane (option 1: tip enriched; option 2: base enriched), and finally tip and base of fin ray (option 1: tip enriched; option 2: base enriched) using Pearson’s Chi-square test of a 2 × 2 matrix. The test was conducted in R [36].

## Results

### Hypothesis 1 –Variation between tip, mid and base sections of composite fin

#### Salmon

**Fig 3:**
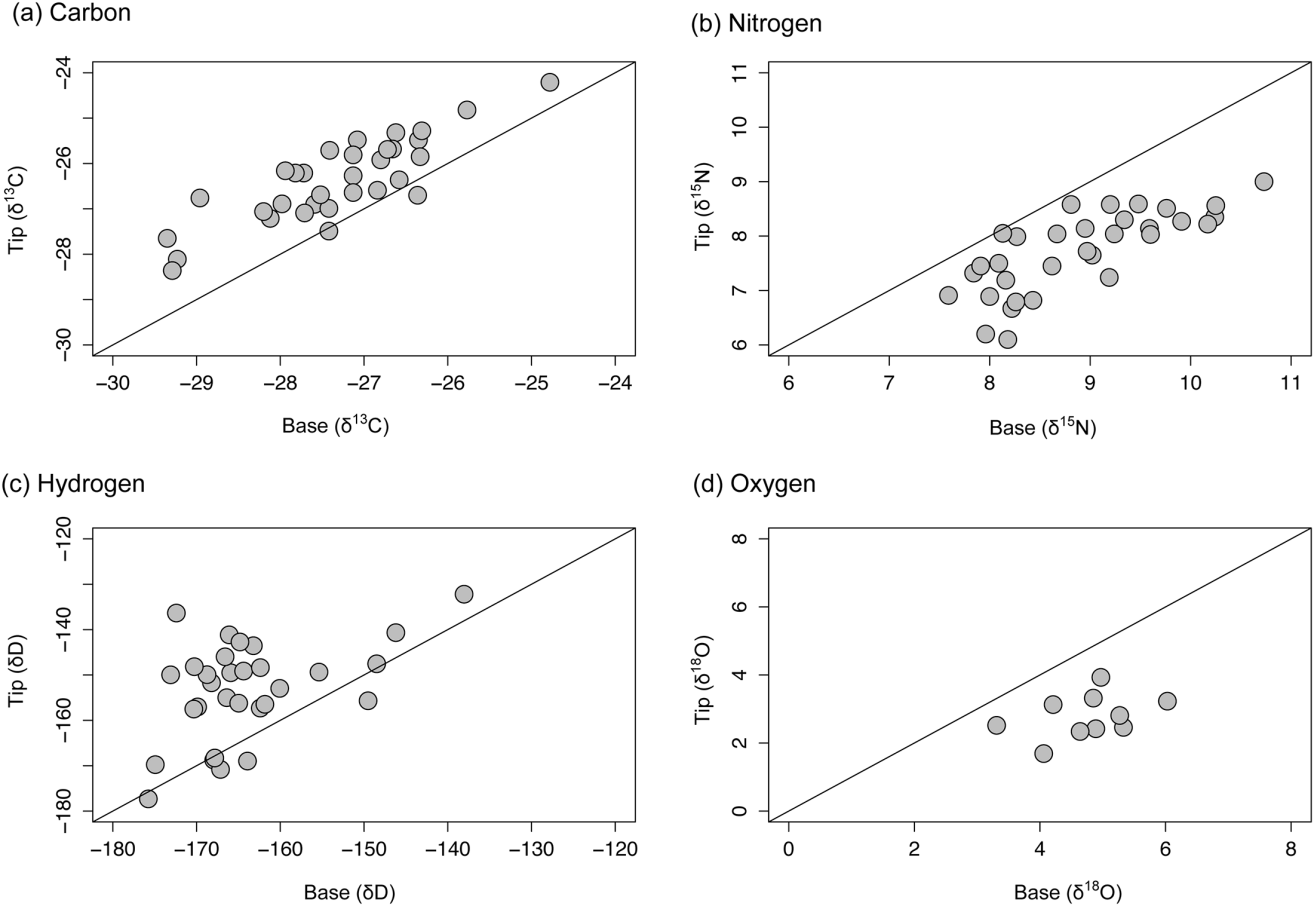
Variation of isotope ratios in salmon fin (TMB). Comparison of the (a) carbon (*δ* ^13^C), (b) nitrogen (*δ* ^15^N), (c) hydrogen (*δ* ^2^H) and (d) oxygen (*δ* ^18^O) stable isotope ratios of base and tip sections of non-treated Atlantic salmon fins. 1:1 lines are added to each plot for visualisation purposes.

**Fig 4:**
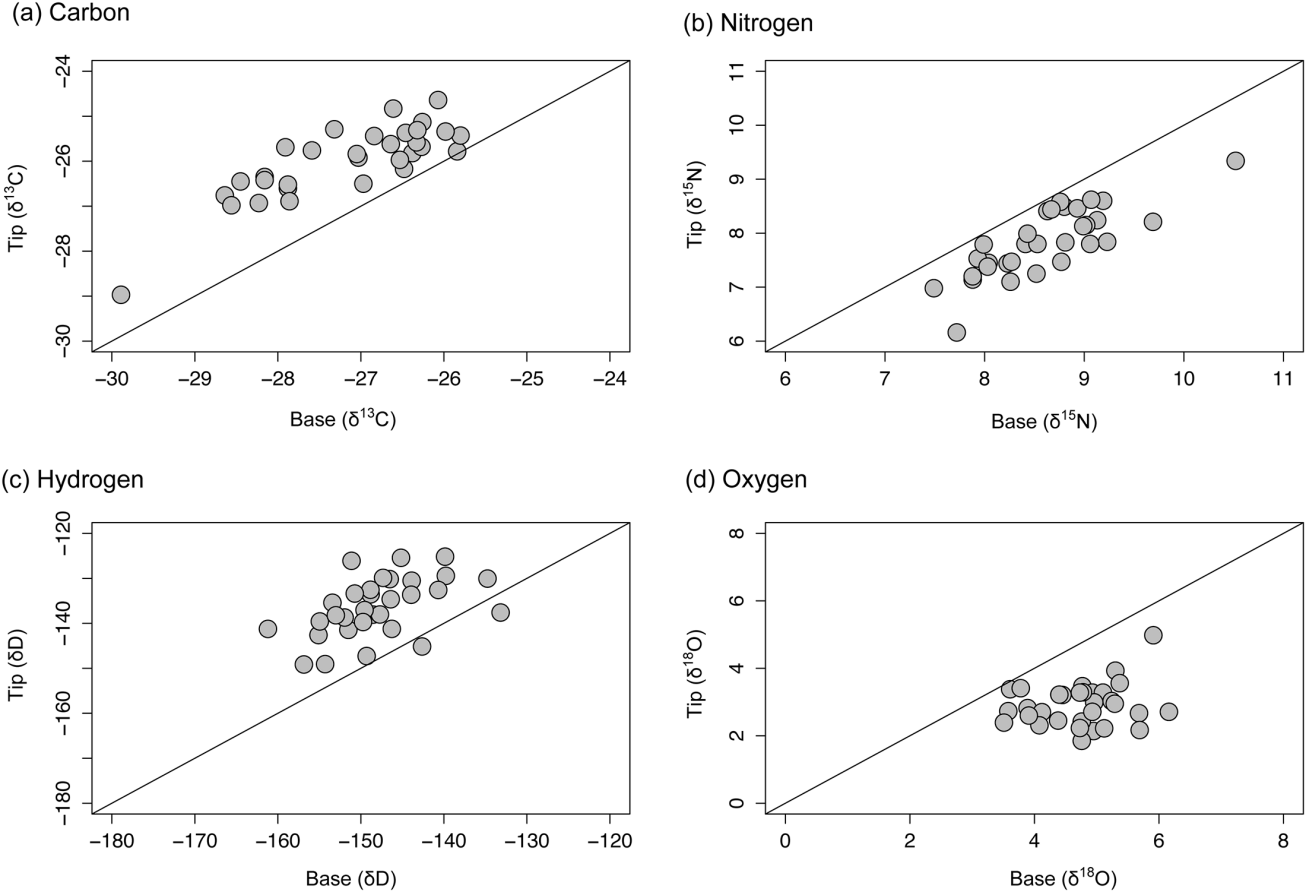
Variation of isotope ratios in lipid-treated salmon fin (TMB.LE). Comparison of the (a) carbon (*δ* ^13^C), (b) nitrogen (*δ* ^15^N), (c) hydrogen (*δ* ^2^H) and (d) oxygen (*δ* ^18^O) stable isotope ratios of base and tip sections of Atlantic salmon fins from which lipids have been extracted. 1:1 lines are added to each plot for visualisation purposes.

In the TMB group, tip sections were ^13^C enriched (1.0 ± 0.6 ‰ SD) relative to base sections (Fig 3a). This variation was equally distributed between the tip, mid and base sections (Table 1). A similar trend was observed in the TMB.LE group; tip sections were enriched (1.2 ± 0.6 ‰) relative to base sections with mid sections intermediate between both extremes (Fig 4a; Table 1). Tip sections were ^15^N depleted relative to base sections in both TMB (1.2 ± 0.6 ‰; Fig 3b) and TMB.LE (0.8 ± 0.4 ‰; Fig 4b) fins. This variation was equally dispersed between the tip, mid and base sections (Table 1). Intra-individual variation in δ^2^H exceeded that evident in the other three isotopes, in the most extreme cases differences of greater than 30 ‰ were observed between tip, mid and base sections of a single fin. On average, tip sections were ^2^H enriched relative to base sections by 10 ± 10 ‰ and 12 ± 6 ‰ in TMB (Fig 3c) and TMB.LE (Fig 4c) samples, respectively. Tip sections were ^18^O depleted relative to tip sections by 1.9 ± 0.7‰ in TMB and by 1.8 ± 0.8‰ in TMB.LE groups (Figs 3d and 4d; Table 1). Mid sections were intermediate between tip and base (Table 1).

**Table 1:** Intra-fin variation in carbon (δ^13^C), nitrogen (δ^15^N), hydrogen (δ^2^H) and oxygen (δ^18^O) stable isotope ratios.

		Tip—Base			Tip—Mid			Mid—Base		
Isotope	n	Mean (SD)	t _(1, n-1)_	*P*	Mean (SD)	t _(1, n-1)_	*P*	Mean (SD)	t _(1, n-1)_	*P*
***Salmon—non treated***										
**δ** ^**13**^ **C**	32	1.0 (0.6)	-9.7	< 0.001	0.4 (0.5)	-4.9	< 0.001	0.5 (0.5)	-5.5	< 0.001
**δ** ^**15**^ **N**	32	-1.2 (0.6)	11.9	< 0.001	-0.6 (0.5)	6.4	< 0.001	-0.6 (0.3)	11.8	< 0.001
**δ** ^**2**^ **H**	34	9.5 (10.3)	-5.38	< 0.001	4.9 (8.5)	-3.4	< 0.001	4.5 (7.5)	-3.5	< 0.001
**δ** ^**18**^ **O**	14	-1.9 (0.7)	9.9	< 0.001	-1.4 (0.8)	6.8	< 0.001	-0.6 (0.7)	3.2	< 0.001
***Salmon—lipid extracted***										
**δ** ^**13**^ **C**	31	1.2 (0.6)	-11.5	< 0.001	0.7 (0.5)	-9.2	< 0.001	0.5 (0.5)	-5.4	< 0.001
**δ** ^**15**^ **N**	31	-0.8 (0.4)	10.7	< 0.001	-0.3 (0.3)	5.2	< 0.001	-0.5 (0.4)	6.4	< 0.001
**δ** ^**2**^ **H**	31	11.6 (6.4)	-10.1	< 0.001	7.4 (4.6)	-9.1	< 0.001	4.1 (4.9)	-4.7	< 0.001
**δ** ^**18**^ **O**	31	-1.8 (0.8)	12.1	< 0.001	-1.1 (0.8)	7.9	< 0.001	-0.7 (0.7)	5.7	< 0.001
***Trout***										
**δ** ^**13**^ **C**	9	0.8 (0.9)	-2.7	0.033	0.8 (1.1)	-2.1	0.072	0.1 (0.6)	-0.1	0.998
**δ** ^**15**^ **N**	9	-1.3 (0.5)	7.2	< 0.001	-0.8 (0.5)	4.8	< 0.001	-0.5 (0.4)	3.6	< 0.001
**δ** ^**2**^ **H**	10	17.6 (7.2)	-7.7	< 0.001	11.2 (7.8)	-4.5	< 0.001	6.4 (5.7)	-3.6	< 0.001
**δ** ^**18**^ **O**	-	-	-	-	-	-	-	-	-	-

Number of fins analysed (n) and mean (‰) difference between tip-base, tip-mid and mid-base comparisons of stable isotope ratios in non-treated (TMB) and lipid extracted (TMB.LE) Atlantic salmon and non-treated anadromous brown trout. Standard deviation of difference is presented in parentheses. Results of paired Welch t-test of the difference is also presented, degrees of freedom for each test are n– 1.

The removal of lipids had no effect on the degree of variation between tip and base sections in carbon (t = 1.52; d.f. = 60.842; P = 0.134), oxygen (t = 0.46; d.f = 28.58; P = 0.647) or hydrogen (t = 1.01; d.f. = 56.11; P = 0.313) stable isotope ratios. Statistically significant variation was evident in stable isotopes of nitrogen (t = 3.31, d.f. = 56.18; P = 0.001) but the actual variation in isotope ratios (mean ± SD; non treated: 1.2 ± 0.6; treated: 0.8 ± 0.4) was small and unlikely to confer any biological significance.

#### Trout

**Fig 5:**
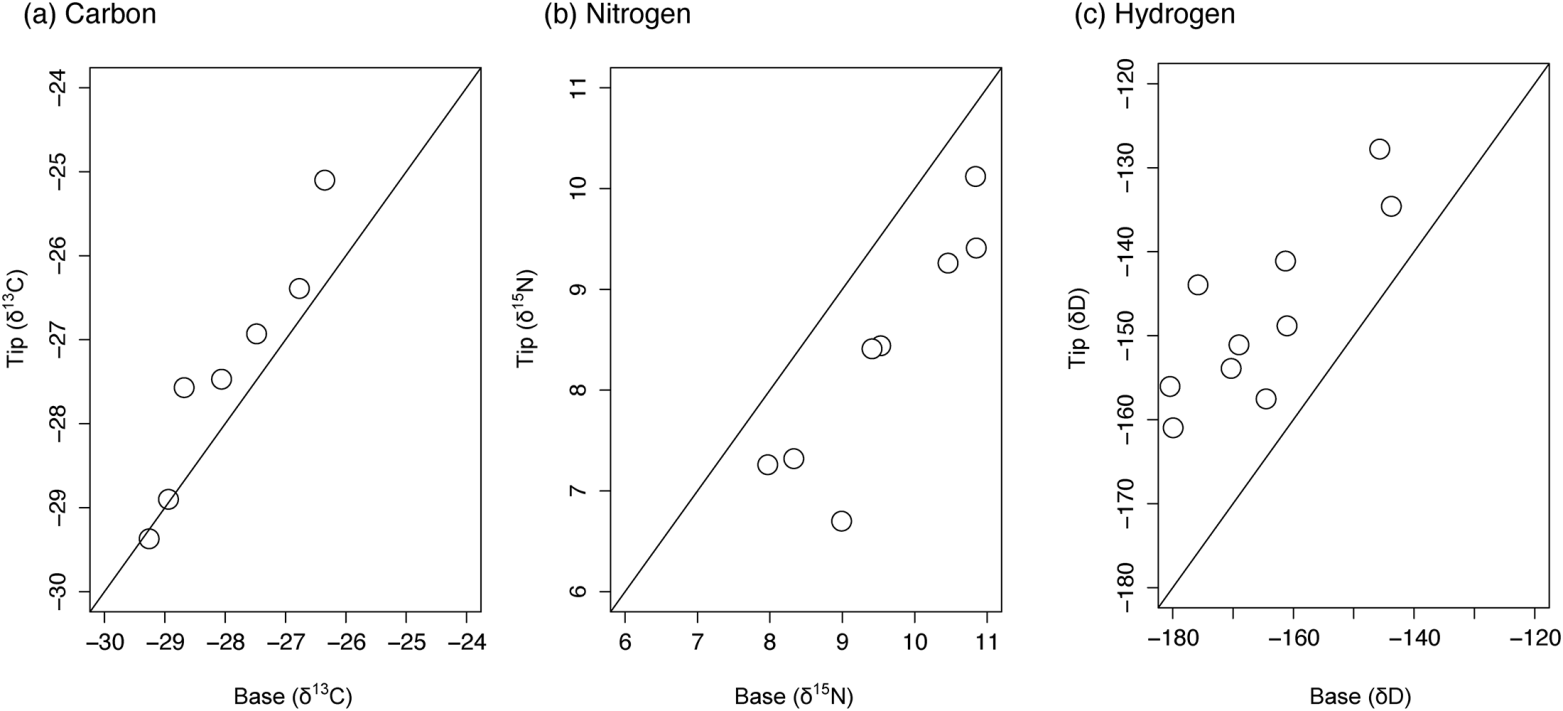
Variation of isotope ratios in trout fin. Comparison of the (a) carbon (*δ* ^13^C), (b) nitrogen (*δ* ^15^N), (c) hydrogen (*δ* ^2^H) stable isotope ratios of base and tip sections of non-treated anadromous brown trout fins. 1:1 lines are added to each plot for visualisation purposes.

The variation in tip-mid-base comparisons of trout mirrored that evident in salmon. Tip sections were ^13^C enriched (0.8 ± 0.9 ‰) and ^15^N depleted (1.3 ± 0.5 ‰) relative to base (Fig 5a–5b; Table 1). This variation was equally dispersed between tip, mid and base sections (Table 1). Tip sections were ^2^H enriched relative to base sections (17.6 ± 7.2 ‰; Fig 5c), with the variation equally split between tip, mid and base comparisons (Table 1). Due to sample size limitations, oxygen isotope ratios of trout fins were not analysed.

### Hypothesis 2 –Variation between ray and membrane

**Fig 6:**
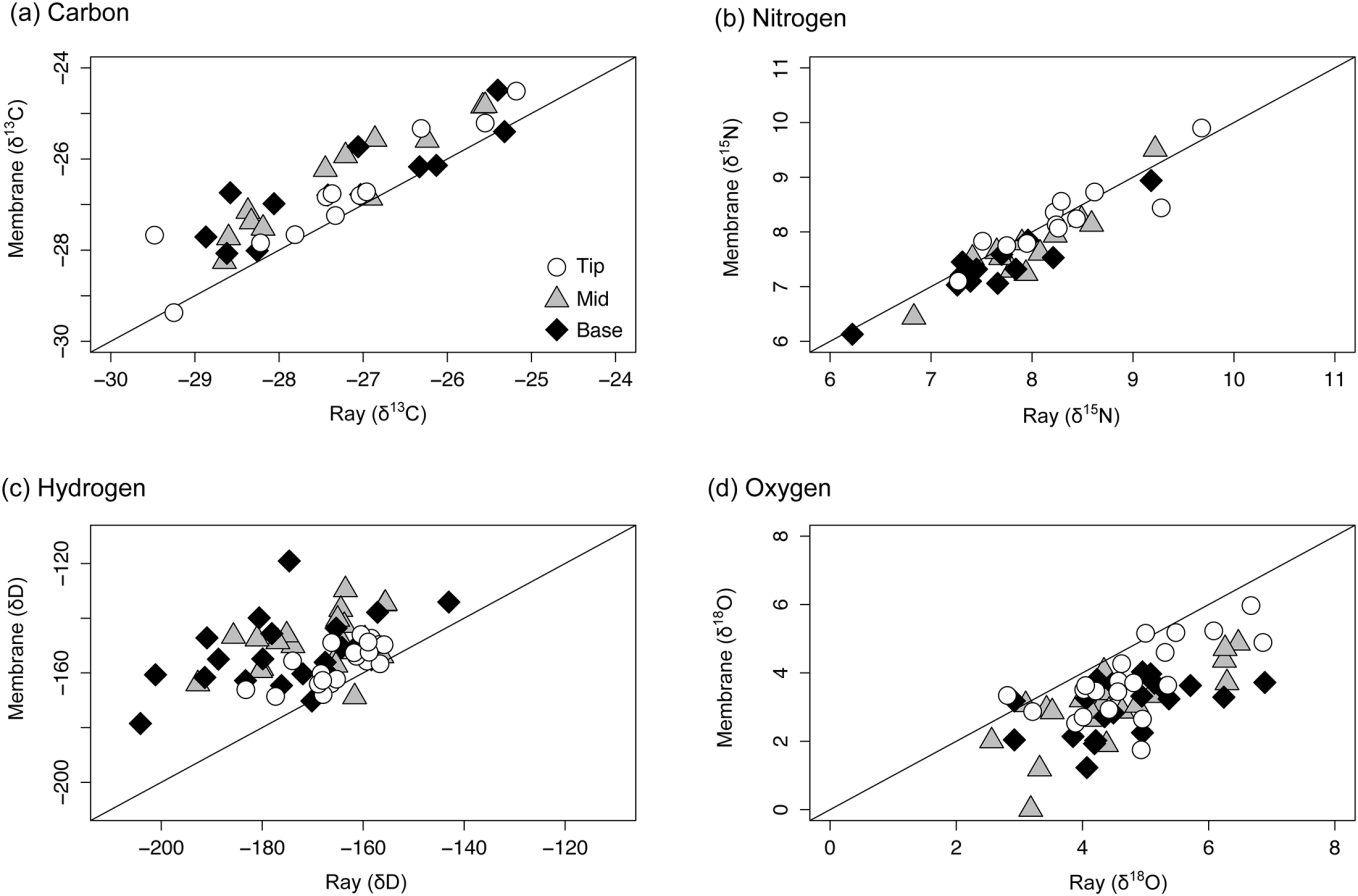
Variation of isotope ratios between fin ray and fin membrane. Comparison of the (a) carbon (*δ* ^13^C), (b) nitrogen (*δ* ^15^N), (c) hydrogen (*δ* ^2^H) and (d) oxygen (*δ* ^18^O) stable ratios of ray and membrane components of tip, mid and base sections of non-treated Atlantic salmon fins. 1:1 lines are added to each plot for visualisation purposes.

The *δ* ^13^C, *δ* ^15^N *δ* ^2^H and *δ* ^18^O isotope ratios of ray and membrane differed in tip, mid and base sections. Fin membrane was ^13^C enriched relative to ray in tip (0.5 ± 0.5 ‰), mid (0.8 ± 0.4 ‰) and base (0.7 ± 0.6 ‰) sections (Fig 6a; Table 2). Variation in δ^15^N values between membrane and ray, though statistically significant (Table 2), was too small to be biologically meaningful (Fig 6b); membrane was ^15^N enriched relative to ray in tip (0.1 ± 0.3 ‰), mid (0.2 ± 0.3 ‰) and base sections (0.3 ± 0.2 ‰; Table 2). Fin membrane was ^2^H enriched relative to ray in all sections (Fig 6c) though the difference was most pronounced in mid (21 ± 11 ‰) and base (22 ± 15 ‰; Table 2) sections. Fin membrane was ^18^O depleted relative to ray (Fig 6d) and this variation was evident in tip (0.9 ± 0.8 ‰), mid (1.4 ± 0.8 ‰) and base (1.7 ± 0.9 ‰) sections (Table 2).

**Table 2:** Variation in carbon (δ^13^C), nitrogen (δ^15^N), hydrogen (δ^2^H) and oxygen (δ^18^O) stable isotope ratios between fin ray and membrane.

		Tip			Mid			Base		
Isotope	n	Mean (SD)	t _(1, n-1)_	*P*	Mean (SD)	t _(1, n-1)_	*P*	Mean (SD)	t _(1, n-1)_	*P*
**δ** ^**13**^ **C**	12	0.5 (0.5)	-3.4	< 0.001	0.8 (0.4)	-7.6	< 0.001	0.7 (0.6)	-3.9	< 0.001
**δ** ^**15**^ **N**	12	-0.1 (0.3)	0.6	< 0.001	-0.2 (0.3)	2.9	0.011	-0.3 (0.2)	3.5	< 0.001
**δ** ^**2**^ **H**	21	8.1 (5.3)	-6.9	< 0.001	20.9 (10.9)	-8.8	< 0.001	22.2 (14.8)	6.9	< 0.001
**δ** ^**18**^ **O**	21	-0.9 (0.8)	5.3	< 0.001	-1.4 (0.8)	7.9	< 0.001	-1.7 (0.9)	8.6	< 0.001

Number of fins analysed (n) and the mean (‰) difference between the isotope ratios of membrane and ray tissues in tip, mid and base sections of Atlantic salmon fins. Standard deviations are presented in parentheses. Results of a paired Welch t-test of the difference between ray and membrane in each section are also provided. Degrees of freedom are n– 1 in all cases.

### Hypothesis 3 –Longitudinal variation in ray and membrane

**Fig 7:**
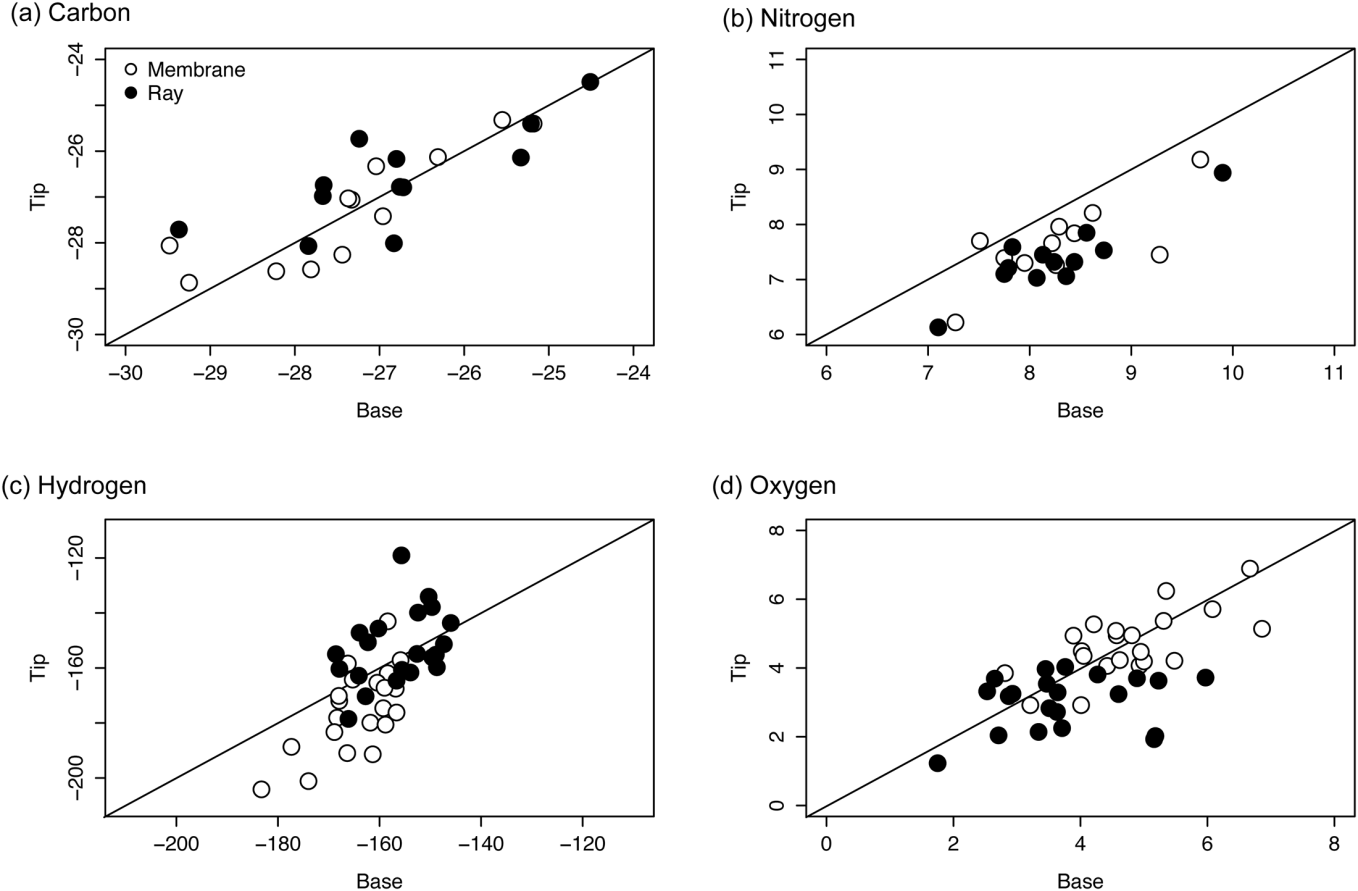
Longitudinal variation of isotope ratios in fin ray and fin membrane. Comparison of the (a) carbon (*δ* ^13^C), (b) nitrogen (*δ* ^15^N), (c) hydrogen (*δ* ^2^H) and (d) oxygen (*δ* ^18^O) stable ratios of tip and base sections of the ray and membrane components of non-treated Atlantic salmon fins. 1:1 lines are added to each plot for visualisation purposes.

When assessed independently, the variation between tip, mid and base sections of fin ray and membrane was minimal, and only evident in certain stable isotope values (Fig 7; Table 3). Variation in δ^13^C values between tip, mid and base sections of both ray and membrane was not significant (Fig 7a, Table 3). Tip sections of membrane (0.7 ± 0.5 ‰) and ray (0.9 ± 0.3 ‰) were ^15^N depleted relative to base sections (Fig 7b; Table 3). This variation was equally dispersed between tip, mid and base sections of both tissues (Table 3). Variation in δ^2^H values was evident between tip, mid and base sections of fin membrane (Fig 7c, Table 3), but not fin ray (Fig 7c). In contrast to the variation observed between tip, mid and base sections of composite fin (TMB), tip sections of membrane were ^2^H depleted (10.6 ± 11.6 ‰) relative to base sections (Table 3). Variation in the δ^18^O values of membrane was not significant (Fig 7d, Table 3), but tip sections of ray were ^18^O enriched relative to mid (0.8 ± 0.9 ‰) and base (0.8 ± 1.2 ‰).

**Table 3:** Longitudinal variation of carbon (δ^13^C), nitrogen (δ^15^N), hydrogen (δ^2^H) and oxygen (δ^18^O) stable isotope ratios in fin ray and membrane.

		Tip—Base			Tip—Mid			Mid—Base		
Isotope	n	Mean (SD)	t _(1, n-1)_	*P*	Mean (SD)	t _(1, n-1)_	*P*	Mean (SD)	t _(1, n-1)_	*P*
***Membrane***										
**δ** ^**13**^ **C**	12	0.1 (0.6)	-0.4	0.74	-0.1 (0.5)	0	1.000	0.1 (0.3)	-0.8	0.401
**δ** ^**15**^ **N**	12	-0.7 (0.5)	4.7	< 0.001	-0.3 (0.4)	3	0.001	-0.4 (0.3)	4.1	< 0.001
**δ** ^**2**^ **H**	21	-10.6 (11.6)	4.2	< 0.001	-4.6 (9.5)	2.2	0.043	-5.9 (13.8)	1.9	0.059
**δ** ^**18**^ **O**	21	-0.1 (0.8)	0.4	0.698	-0.3 (0.7)	2	0.062	0.3 (1.0)	-1.2	0.300
***Ray***										
**δ** ^**13**^ **C**	12	0.2 (0.9)	-0.9	0.400	0.3 (0.6)	-1.8	0.101	-0.1 (0.5)	0.7	0.501
**δ** ^**15**^ **N**	12	-0.9 (0.3)	9.9	< 0.001	-0.5 (0.2)	7.3	< 0.001	-0.4 (0.2)	5.5	< 0.001
**δ** ^**2**^ **H**	21	3.5 (12.5)	-1.3	0.202	8.2 (7.8)	-4.8	< 0.001	-4.7 (12.1)	1.8	0.094
**δ** ^**18**^ **O**	21	-0.8 (1.2)	2.9	< 0.001	-0.8 (0.9)	3.8	< 0.001	0.1 (0.8)	-0.2	0.903

Number of fins analysed (n) and the mean (‰) difference between the isotope ratios of tip, mid and base sections in ray and membrane tissues of Atlantic salmon fins. Standard deviations are presented in parentheses. Results of a paired Welch t-test of the difference between ray and fin in each section are also provided. Degrees of freedom are n– 1 in all cases.

Chi-square tests of carbon and oxygen isotope ratios indicated that variation between tip and base sections was most similar to variation between membrane and ray (Table 4). Tip—base variation in nitrogen in composite (membrane + ray) was significantly different from variation between membrane and ray, but similar to tip—base variation in membrane and ray (Table 4). Tip-base variation in hydrogen differed from membrane ray and tip-base variation of membrane but not from tip-base variation in fin ray. A slight modification of the test, characterising the enriched / depleted cut off at +4 / -4 ‰ respectively rather than 0, which limited the potential error associated with slightly enriched or depleted samples, indicated that tip—base variation in hydrogen was similar to variation between membrane and ray (χ^2^ = 1.555, *P* = 0.212).

**Table 4:** Chi-square tests comparing the variation between stable isotope ratio of tip and base section with membrane and ray and tip and base sections of fin membrane and ray separately.

	Membrane—Ray		Tip—Base (membrane)		Tip—Base (Ray)	
	χ^2^	*P*	χ^2^	*P*	χ^2^	*P*
**δ** ^**13**^ **C**	<0.001	1	5.749	0.016	8.481	0.003
**δ** ^**15**^ **N**	6.274	0.013	0.266	0.606	0	1
**δ** ^**2**^ **H**	7.646	0.006	17.722	<0.001	2.415	0.120
**δ** ^**18**^ **O**	0.005	0.945	8.402	0.004	3.936	0.047

## Discussion

We detailed significant differences in the *δ* ^13^C, *δ* ^15^N, *δ* ^2^H and *δ* ^18^O values of membrane and ray components in the caudal fins of juvenile Atlantic salmon and anadromous brown trout. These differences were sufficient to drive mean variation in excess of 1 ‰ in *δ* ^13^C and *δ* ^15^N, 2 ‰ in *δ* ^18^O and 10 ‰ in *δ* ^2^H between the base and tip section of fins. Our hypothesis, that intra-fin variability would be evident in the caudal fins of salmon and trout, was roundly supported by the data. Furthermore, similar trends were observed in fins that contained lipids and those which had undergone lipid extraction. Our results indicate that the variation was most likely due to differences in the relative abundance of ray and membrane in each fin section. However, fin growth and regeneration appears to be an important factor in relation to *δ* ^15^N.

The variation in *δ* ^13^C, *δ* ^2^H, *δ* ^18^O and to a lesser extent *δ* ^15^N between ray and membrane in tip, mid and base sections echoed the variation between tip, mid and base sections of composite (ray + membrane) fin. Chi-square tests of H isotopes gave contrasting results depending on whether the cut off between enriched and depleted samples as set at 0 or at +4 / -4. We are inclined to favour the second comparison, which takes into account the greater experimental error (~ 2 ‰) and greater range of stable isotope values (~ 100 ‰) associated with stable isotope ratios of hydrogen, and is commensurate with the similar range of variation evident between both comparisons. Fish fin is a heterogeneous structure with a thin membrane layer supported by a fan-like arrangement of rays [29 and references therin]. As a consequence, the ratio of ray to membrane is likely higher in base sections than tip. Fin ray and base sections of composite fins were ^13^C and ^2^H depleted, and ^18^O enriched relative to membrane and the tip sections of composite fins. Furthermore, the degree of variation (1 ‰ in *δ* ^13^C, 1–2 ‰ in *δ* ^18^O and 10 ‰ in *δ* ^2^H values) was equivalent in both comparisons, indicating that the difference between ray and membrane accounted for the differences between base and tip sections. In contrast, variation in these isotope values between tip, mid and base sections of ray and membrane analysed independently was minimal. Variation evident in the *δ* ^2^H values of membrane, whereby base sections were ^2^H enriched relative to tip, ran counter to variation observed in composite fins and therefore is not related to the overall trends. Some variation was also evident in *δ* ^18^O values, though this variation was not evenly dispersed through the fin. Tip sections of fin ray were enriched in ^18^O relative to mid and base sections, indicating that it may relate to a regeneration of fin tips following a seasonal change in the *δ* ^18^O values of the water in the Tornionjoki river system. Previous studies have demonstrated that the oxygen isotopic composition of river water vary between seasons [37], but further work within this region is required to ascertain whether such seasonal variation in the Tornionjoki River could be a driver of the variation in isotope ratios recorded here.

Variation in *δ* ^15^N values ran counter to the trend observed in *δ* ^13^C, *δ* ^2^H and *δ* ^18^O. Variation between ray and membrane was minimal, but tip sections of both ray and membrane were ^15^N enriched relative to base section, mirroring the trend in composite fins. As such the variation in *δ* ^15^N detailed in fish fins is likely related to growth or the regeneration of tissue rather than variation between ray and membrane components of the fin. Salmon smolts in the Tornionjoki River predominantly feed on Perlodidae larvae [38] which are predators of other benthic macroinvertebrates [39]. An increased gape size, associated with smoltification, would enable salmon smolts to forage on larger Perlodidae larvae than parr potentially resulting in the observed increase in *δ* ^15^N. In any case, the variation evident in *δ* ^15^N and *δ* ^18^O values indicates that variation between fin sections cannot solely be considered in terms of ray and membrane and may also be affected by growth and regeneration.

The principal objective of this study was to examine the variation of isotope ratios within fins. As such, we do not have sufficient information to conclusively determine the causes of the variation in isotope ratios between fin ray and membrane. Further experimental studies in controlled environments would be required to assess this. Our observations, however, provide an opportunity to speculate about the mechanistic processes. Fin ray is composed of bone (calcium phosphate) and collagen [29], and thus may be ^13^C enriched due to the presence of carbonates [27,40]. However, ray was ^13^C depleted relative to membrane indicating that bias due to carbonates is not an issue. Rather, we suggest that difference in turn-over rates between ray and membrane may account for the variation evident in isotope ratios. Bone collagen has a longer isotope turnover rate than most tissues [41,42]. Therefore, the isotope ratios of fin ray likely reflect a longer period in the life history of a fish than membrane [10]. The fish analysed in this study were collected during the early summer months. As such, their isotope ratios likely reflected their diet and habits during the winter and spring. In northern Finland, this time period is associated with a shift from 24-hour darkness, ice cover and low autochthonous productivity to 24-hour sunlight and high productivity [43]. Associated with this is a large influx of meltwater which may have altered the *δ* ^2^H and *δ* ^18^O values of the river [44,45]. Against this backdrop of variation in the isotope ratios of prey and environment, differences in the turnover rate of ray and membrane would likely result in significant differences in the isotope ratios of each tissue. Further studies in this region incorporating a seasonal dimension are required to determining the seasonal variation in isotope ratios of water and consumers[43].

The potential effect of lipids on our results warrants further attention. Lipids are depleted in ^13^C and ^2^H relative to protein and previous studies have indicated that failing to remove lipids prior to analysis can incorrect estimation of the isotope ratios of fish fin [27]. Estimations of *δ* ^2^H values for tissues containing lipids are further complicated as the fraction of exchangeable H with ambient vapour can differ between tissues, potentially biasing these measurements [33]. Although we analysed treated and non-treated samples, we did not have sufficient material to analyse the same samples before and after lipid treatment. Thus we cannot categorically account for the effect of lipids in the TMB and TMB.RM groups. A preliminary study from the same fish fin samples that were analyzed before and after lipid extraction (Soto et al., unpublished data) indicated that lipid removal resulted in an enrichment of, on average, 25.8 ‰ (± 11.5 SD) in each sample and reduced the variation in *δ* ^2^H between tip and base sections by approximately 50%; however, this study had a limited sample size (n = 6). As such, we recommend that lipid removal be included in the standard sample preparation procedures for fish fin [27] but contend that further, non-lipid related variation is also evident within fish fin.

The levels of intra-individual variation detailed here are sufficient to cause concern for researchers whose work is entirely based on fin clips. For example, this study resulted from a failed attempt to use stable isotopes to conduct a mixed stock analysis of origins of salmon and trout smolts in the Tornionjoki River system. We intended to use stable isotope mixing models to relate the isotope ratios of juvenile salmon and sea trout in potential natal streams to the isotope ratios of sea-going smolts. Doing so would have revealed the relative contribution of different natal streams to the overall population of salmon and trout in the river. However, the variation within individual fish exceeded the variation among juvenile fish in natal streams throughout the catchment, rendering the initial investigation ineffective. The variation evident in *δ* ^13^C, *δ* ^15^N and *δ* ^18^O values exceeded 2 ‰ in many fish. While not sufficient to dramatically alter inferences regarding a fishes’ trophic level or resource use, subtle differences in this range have been associated with variation between individuals within a population or an individual’s placement upon a scale of resource use [46,47]. Furthermore, if the variation between tip and base sections relates to temporal variation in resource use then this temporal variation would be masked when conducting an analysis of composite fin tissue. The variation evident in *δ* ^2^H between fin sections far exceeded that of other isotopes. In the most extreme cases, *δ* ^2^H ratios within individual fins varied by over 30 ‰. Given that the entire range of δ^2^H values recorded in the study was approximately 40 ‰, this variation within a fin essentially covered the entire range of *δ* ^2^H values within the catchment. This finding raises some concerns regarding the suitability of fish fin as a medium for δ^2^H analysis, especially for studies aimed at identifying migration patterns or natal ranges. Further testing of intra-fin variability in other catchments and with other species will be required to establish the suitability of *δ* ^2^H values of fish fin as a marker for migration in freshwater fishes. It is worth noting, however, that the typical variation between fin sections was approximately 10 ‰. When considered in relation to the analytical precision of *δ* ^2^H measurements compared to other isotopes studied here, this degree of variation is comparable to that reported for *δ* ^13^C, *δ* ^15^N and *δ* ^18^O values.

The intra-individual variation evident in stable isotope ratios of fin may also be relevant to studies using other tissues. Although the most common matrices for stable isotope analysis (muscle, liver, hair, bone, vibrissae and keratin [1]) are homogenous materials in comparison to fish fin, the potential for intra-individual variability in these tissues has rarely been studied. One such study in Atlantic salmon, identified variation exceeding 1 ‰ in the *δ* ^13^C and *δ* ^15^N values of muscle tissue from an individual fish [48]. The variation in *δ* ^13^C was likely related to lipid reserves but the variation in *δ* ^15^N indicates that additional mechanisms may be responsible for determining the isotope ratios in specific tissues. In addition, as stable isotope turnover rates are inextricably linked with growth [10,11] it is reasonable to hypothesise that active muscle tissues undergoing growth or repair may have different stable isotope ratios than inactive muscle tissues.

Our results indicate that further studies into the variability within fins should be conducted prior to the replacement of traditional tissues such as muscle by fish fin. Numerous controlled studies have been conducted to determine the isotopic turnover rate of various tissues, including fin [10,49]. However, to the best of our knowledge no study has explicitly tested the difference between fin membrane and ray. Until the variation between ray and membrane is well quantified we propose three options to facilitate the continued use of non-lethal fin samples: 1) researchers using fin clips should also sacrifice a small number of fish from which isotope ratios of tissues with a known turnover time (e.g. liver and muscle) can be obtained. These values may act as a correction factor for fin clips allowing researchers to relate the isotope ratio of fins to homogenous tissues with an established turnover rate and identify any potential bias associated with the fin clips. 2) Fin clips should be restricted to the tip section, decreasing the variation in the amount of ray in the sample. 3) If options 1 or 2 above are not feasible, fin clips may be homogenized to provide a uniform value representing the full fin. However, this option may also be prone to bias as the composite value will be a reflection of the proportion of ray and membrane in the sample.

In conclusion, we have identified consistent and ecologically significant variation in the *δ* ^13^C, *δ* ^15^N, *δ* ^2^H and δ^18^O values of the caudal fins of Atlantic salmon and anadromous brown trout. This variation is predominantly related to the difference in isotope ratios of ray and membrane components, but additional variation associated with fin regeneration and lipid reserves are also evident. These variations should not dissuade researchers from using non-lethal fin clips as an alternative to tissues that require lethal sampling, but they do demonstrate a need for researchers to account for this variation when sampling, treating and preparing fins for stable isotope analysis.

## Supporting Information

S1 File Stable isotope values. A data file containing all carbon (δ^13^C), nitrogen (δ^15^N), hydrogen (δ^2^H) and oxygen (δ^18^O) stable isotope ratios generated during this study.
(XLSX)
